# The Hospital World

**Published:** 1910-11-26

**Authors:** 


### The Hospital World.

### Personalia.

His Majesty the King has consented to become patron of the Salford Royal Hospital.

Prince Alexander of Teck has been informed by the students of the Middlesex Hospital Medical School that at a general meeting of students held on Friday, November 18, it was unanimously resolved that past and present students, being anxious to show their grateful appreciation of the great work which the late Prince Francis of Teck did on behalf of the hospital and its school, should raise among themselves 1,000 guineas for the endowment of a Prince Francis Memorial Bed. Prince Alexander has been much encouraged by this further proof of practical sympathy with the objects of the Prince Francis of Teck Memorial Fund, more especially as it came from those who had been able to see and appreciate the value of his brother's work.

One of the first women in the country, and, we are delighted to add, an institutional worker, to be offered the post of mayor is Mrs. Lees, whom the citizens of Oldham have thus chosen to honour. We feel sure that no section of the population of Oldham will be more hearty in its expressions of congratulation than the workers in and supporters of the Oldham Infirmary. To them Mrs. Lees has long been well known, and has created a record permanent for posterity in the private home for nurses which she has established and in the numerous improvements which the Infirmary has been able to accomplish by means of her generosity. As an Oldham citizen, a good word the flavour of which the cosmopolitanism of modern life has done much to destroy, Mrs. Lees has held a prominent position. As the wife of the late Mr. Lees, the head of a great firm of textile machinists of that name, she realised the responsibilities of her position, and threw herself into the communal—for the adjective "social" has been appropriated by Mayfair to our lasting loss—life of the town by entering the Town Council in 1907. She has been admitted to the freedom of the borough, with the rare distinction of being the first of both sexes to receive that honour, and thus enjoys the hall-mark of sterling value, the highest dignity that a city can give. Mrs. Lees has carried her good citizenship into almost every activity of the borough, and there are few active social bodies which have not benefited at her hands. The Oldham Infirmary may congratulate itself on having the opportunity of seeing the mayoralty of the city pass into the hands of an old and tried friend.

The annual meeting of the Doncaster Infirmary last week was enlivened by the presentation made by Mr. R. E. Clark, Chairman of the Board of Management, to Dr. Willey on his retirement from the post of senior house surgeon after twenty years' work. The following is the gist of Mr. Clark's very happy speech on the occasion: "My feelings are divided between the pleasure of carrying out a resolution which is very agreeable to all present and the great regret at losing the valuable services of Dr. Willey, who has been with us about twenty years. I have heard repeated testimony borne to his exceeding kindness to the poor. One department of the work he has given special attention to—the out-patient department—where he has been accessible at all times to the poor. I have heard of his kindness to children of very tender years, to whom medicine is seldom very agreeable! The time has come, however, when these services must cease, and the doctor has come to the conclusion that he will embark upon private practice in the town. I can wish him only a happy, successful, and even a distinguished career. So far as the governors of this institution are concerned, without any canvassing or any trouble, as soon as it was decided to present a testimonial to the doctor the money came in freely, and I have pleasure in presenting him with a note for £50." For a summary of the annual meeting and report our readers are referred to the Institutional Notes of last week.

St. Mary's Hospital, Plaistow, E., is about to sustain a great loss in the resignation of its Secretary, Mr. Percy Glenton, who severs his connection with the hospital on December 4. For nearly eleven years Mr. Glenton has held the official position of Secretary, and in that time, from being a poor and unimportant hospital, St. Mary's has become one of the important London women's and children's hospitals, and it is mainly due to the Secretary's energy and work that this change has been brought about. Mr. Glenton was appointed to the secretaryship in January 1900, coming from the East London Hospital for Children at Shadwell, where he had been assistant to the secretary at that institution for four years. At the time of his taking office at St. Mary's the building fund stood at £700. The hospital was in such a deplorable state that Mr. Glenton determined to increase this fund and gather together enough money to build new premises. He steadfastly set to work to accomplish that end, with the result that to-day the fund stands at £14,000 and a new hospital has been built. Again, the amount of money available for endowments was but £400, and this, too, Mr. Glenton determined to increase, and to such good purpose has he given this matter attention that the endowment fund is now £8,317 11s. 9d. From the time of his coming to St. Mary's Hospital the institution has never been in debt. And now, owing to ill-health and personal reasons, he has found it imperative to resign the Secretaryship. The matron (Miss Ray) is getting up a testimonial to present to him, and if by any chance this paragraph should be seen by any who have been residents or in any way connected with the staff of the hospital, or who are interested in the work that is being done by the institution, we feel sure that they would wish to testify their appreciation of Mr. Glenton's good work by sending their subscriptions to the testimonial fund, and so help to make the amount raised worthy of such a good cause. We wish the matron all success in her endeavours. Mr. Glenton deserves the most heartfelt thanks from all connected with St. Mary's, and will carry away with him the sincerest good wishes for his future from all who have come into contact with him, for his courtesy and kindliness have won him many friends during his tenure of office, and his resignation will mean a loss to the institution which will be severely felt.

The Winton and Moordown Hospital Fund, Bournemouth, has had a Chairman and officers who have managed to return nearly £100 to the Bournemouth Fund this year, in spite of a press of difficulties. This was an increase on the previous year's total, which, considering the circumstances, is quite remarkable. The names of the Chairman and his active Committee are: Chairman, Mr. A. Charlo; Vice-Chairman, Mr. T. Tiller; Chairman of Saturday Committee, Mr. Hiscock; Chairman of Sunday Committee, Mr. T. Tiller; Chairman of Fête Committee, Mr. B. Godfrey; Sports Secretary, Mr. A. E. Hoare; Treasurer, Mr. H. Mitchell; Secretary, Mr. L. F. N.

Nash. The Committee is very grateful to the ex-Mayor of Bournemouth, Alderman G. E. Bridge, for his invaluable help.

By direction of Prince Alexander of Teck, Mr. Reginald Lucas has written a short memoir of Prince Francis of Teck, setting forth his services to the Middlesex Hospital. The book will trace Prince Francis's career up to the time when he undertook the work especially connected with his memory, and will be published at once by Mr. Arthur Humphreys, Piccadilly. The profits from its sale will be given to the Prince Francis of Teck Memorial Fund at the Middlesex Hospital.

Last week Viscount Goschen, the chairman of Guy's Hospital, unveiled a memorial bust of the late Dr. Horrocks in the medical school buildings attached to the hospital. The bust, which is of bronze, is at present placed in the entrance lobby to the new physiological department, and on its polished marble pedestal forms a striking decoration for the bare unfurnished little hall. It is a fairly good representation of the late senior obstetric surgeon's features —perhaps modelled in too reposeful an attitude to be altogether satisfactory to those who remember Horrocks as a genial, smiling lecturer, brimming over with good humour, vivacity, and fun. The work of Mr. John Cassidy, it bears the simple inscription :—

Peter Horrocks, M.D.
Obs. Phys. 1883-1909.
Born 1853. Died 1909.

The man whom it represents was one of the best-known members of the Guy's staff, whose reputation as an Alpine climber was as great as his eminence as a gynæcologist. He taught generations of medical men, and not only they who were his old pupils, but the large circle who came into personal contact with him and appreciated his solid qualities, will be glad to find that his bust has now been added to the long gallery of notable physicians and surgeons that look down upon, or up to, the visitor who strolls into the Wills Library.

The supporters, patients, doctors, and nurses in any way connected with the Axminster Cottage Hospital will be glad to see that the annual report, just issued, pays a well-earned tribute of thanks and appreciation to Mr. R. Cornish, J.P. For fourteen years he held the post of hon. secretary, and his retirement in the summer was a loss to the hospital and a break in its history. It was not made less so by the fact that the matron had resigned early in the year, and that the hon. auditor has since done so. Mr. Cornish was succeeded by Mr. A. Hacon, who has taken up the task of carrying on the hospital, whose very existence has been at touch-and-go more than once.

### THE SATURDAY MOVEMENT AT LEICESTER.

The Saturday Hospital Movement at Leicester is growing fast. At a meeting of the Melton Mowbray Committee of the movement it was decided to form a local branch of the Leicester and County Saturday Hospital Society, the parent society having consented to invest such a branch with the full powers of an executive committee. The committee for the local branch will consist of a secretary and twelve members, six of whom are to be the original founders of the local society, not subject to re-election. The remaining six are to be elected at a special meeting of the representatives of subscribing firms called for the purpose. Three of the elective members are to retire annually and to be eligible for re-election.

### Elections and Appointments.

The Rev. G. A. Johnston is the new president of the Grange Hospital Committee, Aberdeen.

Mr. A. Hitchcock has been added to the committee of the Axminster Cottage Hospital, of which Mr. H. Dawkins has been elected hon. auditor for the year.

Mr. H. Stuart Girvan has been re-elected Honorary Secretary and Treasurer, and A. M. Gourlay, C.A., Honorary Auditor of the Glasgow Hospital for Women.

Mr. S. Cornfield has been elected chairman of the Hospital Saturday Committee of the Wolverhampton and Midland Counties Eye Infirmary, in the room of Mr. T. Capper, who has resigned.

Councillor George Chitham (deputy mayor) has been elected a member of the Board of Governors of the Leicester Infirmary in the place of the late Mr. Alfred Colson. Councillor Chitham, during his mayoralty, took an especial interest in the philanthropy of the town, and his services to the Leicester Infirmary were much valued.

Mr. George Blair, Mr. A. L. Harber, and Mr. F. Williamson have been appointed three trustees on the board of directors of the Longton Cottage Hospital, Staffordshire, and the following nine other directors were also elected : The Mayor of Stoke-on-Trent, Mr. J. R. Haines, Mr. G. Nixon, Mr. T. Copestake, Mr. A. Hewitt, Mr. F. A. Spruce, Mr. A. B. Jones, and Mr. A. Parkes, together with a member jointly nominated by the Florence Coal and Iron Company and the Stafford Coal and Iron Company.

